# A citizen science report—Tiger mosquitoes (*Aedes albopictus*) in allotment gardens in Graz, Styria, Austria

**DOI:** 10.1007/s00436-023-08106-9

**Published:** 2023-12-30

**Authors:** Julia Reichl, Christina Prossegger, Bernhard Eichholzer, Pamina Plauder, Maria Sophia Unterköfler, Karin Bakran-Lebl, Alexander Indra, Hans-Peter Fuehrer

**Affiliations:** 1 Institute for Medical Microbiology & Hygiene, AGES - Austrian Agency for Health and Food Safety Ltd., Währinger Straße 25A, 1090 Vienna, Austria; 2 Institute of Parasitology, University of Veterinary Medicine Vienna, Veterinärplatz 1, 1210 Vienna, Austria; 3 Heimgartenverein Schönau, Kasernstraße 60, 8010 Graz, Austria

**Keywords:** *Aedes albopictus*, Graz, Invasive mosquitoes, Austria

## Abstract

*Aedes albopictus*, the Asian tiger mosquito, is an invasive species not native to Europe. Due to its ability to transmit pathogens, such as dengue, chikungunya and Zika viruses, *Ae. albopictus* is considered a major health threat. In Austria, it was first reported in 2012 in the Western province of Tyrol and was documented in the metropolitan area of Vienna in 2020, demonstrating its ability to colonize urban areas. In July 2021, a garden owner from Graz, Styria, Austria, contacted experts because of the possible presence of tiger mosquitoes in an allotment garden complex. Accordingly, citizen scientists collected adult mosquitoes and set up ovitraps. Adults and eggs were sent to the laboratory for morphological examination and molecular DNA barcoding within the mitochondrial *cytochrome c oxidase subunit I* gene. In total, 217 eggs of *Ae. albopictus* were found at the allotment garden as well as at a second location in the city of Graz. In addition, 14 adult *Ae. albopictus* specimens, of which 7 were molecularly identified as an identical haplotype, were collected at the allotment garden. With its mild climate and numerous parks and gardens, Graz provides the perfect environment for reproduction of tropical/subtropical alien *Aedes* mosquitoes. The presence of eggs and adult specimens in the current study period indicates that *Ae. albopictus* is already breeding in Graz. However, monitoring efforts need to be continued to determine whether stable populations of *Ae. albopictus* can survive there.

## Introduction

The introduction and spread of alien mosquitoes in Europe have become a major public health threat (Medlock et al. 2012; Rezza 2016). Mosquitoes of the genus *Aedes* are of particular concern due to their ability to transmit a variety of pathogens relevant for human (Medlock et al. 2015; Paixao et al. 2018) and animal health (Cancrini et al. 2003; Medlock et al. 2012). As of 2022, three (potentially) invasive mosquitoes have been reported in Austria (Bakran-Lebl et al. 2022): the Asian bush mosquito (*Aedes japonicus*), the Korean bush mosquito (*Aedes koreicus*) and the Asian tiger mosquito (*Aedes albopictus*), with the latter being the most relevant alien mosquito for public health (Rezza 2016).

*Aedes albopictus* (Skuse, 1894) originates from the subtropical and tropical Asian-Pacific regions (Paupy et al. 2009) and is the most invasive mosquito species. In the last decades, it has spread from its native region to all other continents except Antarctica (Bonizzoni et al. 2013) and has thereby become a major concern for public health (Rezza 2016). Since the first report of *Ae. albopictus* in Europe in 1979 in Albania (Adhami and Reiter 1998), it has spread to more than 25 European countries with established populations in at least 21 countries (ECDC 2022; Medlock et al. 2015).

In Austria, the Asian tiger mosquito was first reported in 2012 in Tyrol (Seidel et al. 2012). In the following years, this species was identified mainly along motorways in the western and southern parts of Austria (Fuehrer et al. 2020; Schoener et al. 2019). In 2020, *Ae. albopictus* was first found in the city of Vienna (Bakran-Lebl et al. 2021), demonstrating the introduction of this mosquito species to urban areas. Here, we report the first finding of the Asian tiger mosquito in Graz, the second largest city in Austria and the capital city of the province of Styria.

## Material and methods

**Fig. 1:**
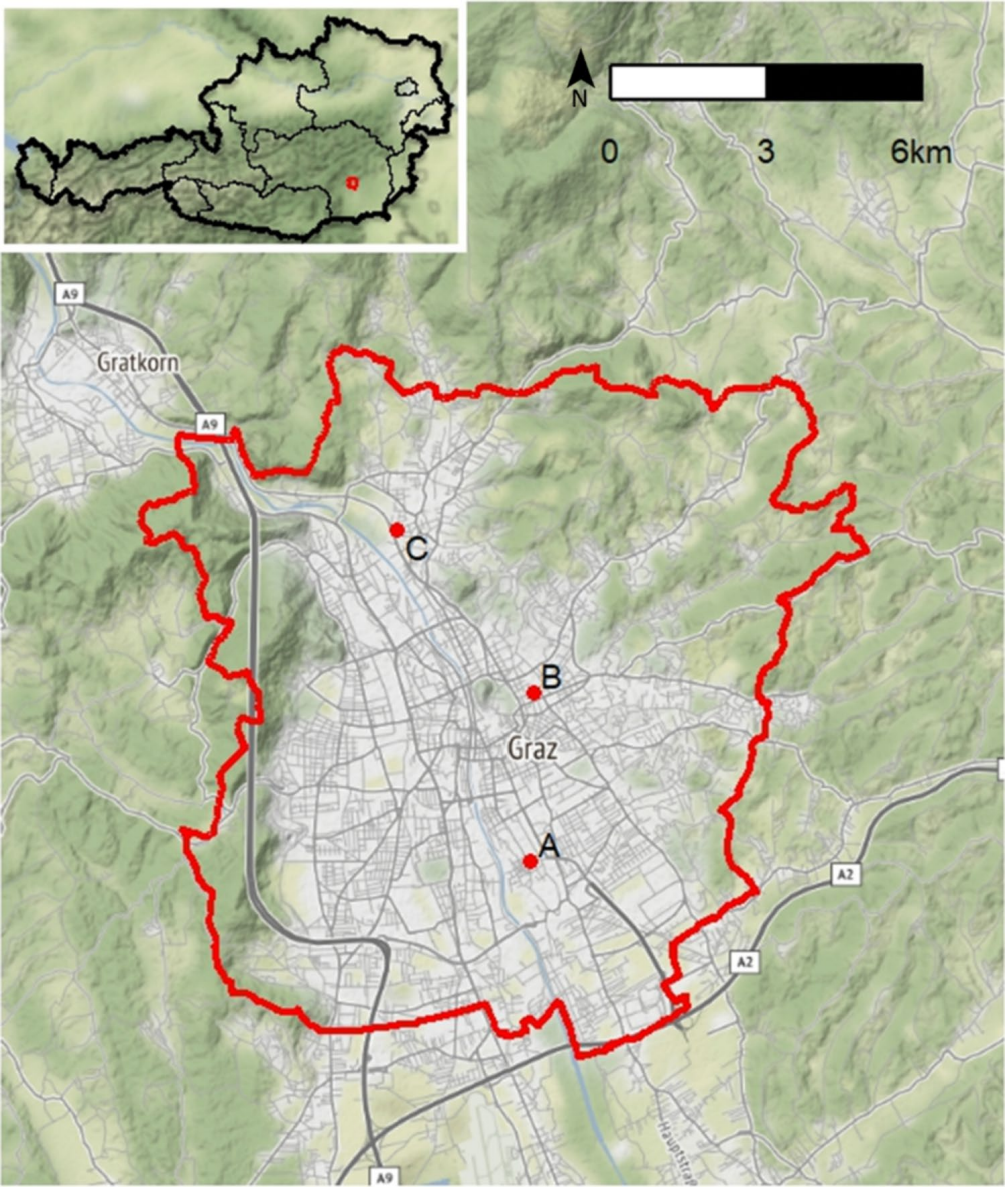
Locations of the sampling sites in Graz, Austria. For monitoring purposes, ovitraps were set up at the allotment garden (**A**), the AGES Institute of Medical Microbiology and Hygiene Graz (**B**) and an industrial park (**C**). The map was drawn using the software R version 4.1.1 (R Core Team 2021) and Map tiles by Stamen Design (Stamen, San Francisco, USA)

In July 2021, a garden owner from Graz, the capital city of the province of Styria, contacted the Institute of Parasitology of the Veterinary University of Vienna and the Austrian Agency for Food and Health Safety (AGES) in Vienna to inform them of the possible presence of tiger mosquitoes in an allotment garden in Liebenau, a municipal district of the city of Graz (47.04911°N, 15.44657°E). In addition, residents of this allotment garden complained because of daytime nuisance caused by mosquitoes, indicating the presence of adult *Aedes* mosquitoes. Accordingly, ovitraps were set up at four different positions at the allotment garden (location A) to check for breeding *Aedes* invasive species (Fig. 1). In parallel, two other locations in Graz (location B: the AGES Institute of Medical Microbiology and Hygiene Graz; location C: an industrial park in Graz) were already being monitored with five ovitraps per location during a nationwide project (Bakran-Lebl 2022). The traps were set up and operated by citizen scientists (location A and C) and employees of the AGES (location B), as described previously (Bakran-Lebl et al. 2022).

Where possible, the individual traps at the respective locations were set up at least 50 m apart from each other to decrease competition between the traps. All ovitraps consisted of a black container (size: 1000 ml) filled with 750 ml tap water. To provide an oviposition possibility, an oviposition substrate (wooden spatula) was inserted into the water and fixed with a steel clamp. Sampling with ovitraps at the allotment garden (location A) started at the beginning of August, while sampling at locations B and C started at the end of April. Monitoring of all three locations ended in the middle of October. During this time, the traps were checked weekly (with one exception where the traps at location C were checked after 2 weeks). During each monitoring round, the oviposition substrate was removed from the container and sent to the laboratory for inspection. The tap water was exchanged, and a new oviposition substrate was positioned in the container for the next round of sampling. In total, 284 oviposition substrates from the three locations (location A:* n* = 29, location B: *n* = 130, location C: *n* = 125) were collected over the course of 26 weeks for locations B and C and over the course of 11 weeks for location A.

In addition, adult mosquitoes were collected by citizen scientists in the allotment garden (location A) and sent to the Institute of Parasitology, Vienna.

Oviposition substrates from ovitraps and adult mosquito specimens were inspected under a stereo microscope for the presence of eggs and morphological specification. Eggs from each oviposition substrate were transferred to an individual Eppendorf tube (1.5 ml) and stored at -80°C until molecular analysis with DNA barcoding of the mitochondrial *cytochrome c oxidase subunit I* gene. In the case of adult mosquitoes, one leg was removed and stored at the same conditions. For analysis, samples were first homogenized on a TissueLyser II (Qiagen, Hilden, Germany) with one ceramic bead (2.8 mm Precellys Ceramic Beads, VWR, Darmstadt, Germany). Subsequently, DNA was extracted using the Qiagen DNeasy Blood&Tissue kit (Qiagen, Hilden, Germany). The protocol and the primers LepF1 and LepR1 were used for PCR of mosquito egg samples, as previously reported (Bakran-Lebl et al. 2022; Hebert et al. 2004). PCR of adult mosquito samples was performed with the primers H15CuliCOIFw and H15CuliCOIRv (Zittra et al. 2016). The resulting PCR amplicons were sent to LGC Genomics (Berlin, Germany) for Sanger sequencing. Subsequently, the sequences obtained were compared to sequences of two publicly available databases, NCBI GenBank (www.ncbi.nlm.nih.gov/genbank, accessed on 10 March 2023) and BOLD Systems (www.boldsystems.org, accessed on 10 March 2023).

For haplotype determination, the sequences of seven adult mosquitoes were compared to the haplotype network of Viennese *Ae. albopictus* samples (Bakran-Lebl et al. 2021) and uploaded to NCBI GenBank (Accession numbers: OR038324 – OR038330) and BOLD Systems (Process IDs: PAVEA244-23 – PAVEA250-23).

## Results and discussion

In total, 284 oviposition substrates were collected from ovitraps, which had been set up by citizen scientists and the AGES at 14 positions at three locations. Over the course of the sampling period, 217 eggs of *Aedes albopictus* were found on a total of 19 oviposition substrates deriving from location A (*n* = 18) and location B (*n* = 1). The eggs of 12 of those 19 oviposition substrates were identified by morphology and molecular analysis. Identification of the eggs of the remaining 7 oviposition substrates was based on morphology alone due to poor quality of the barcoding result.

At the allotment garden (location A), eggs of *Ae. albopictus* were found from the beginning of August until the beginning of October. The single positive sample from location B was collected in mid-June.

**Fig. 2:**
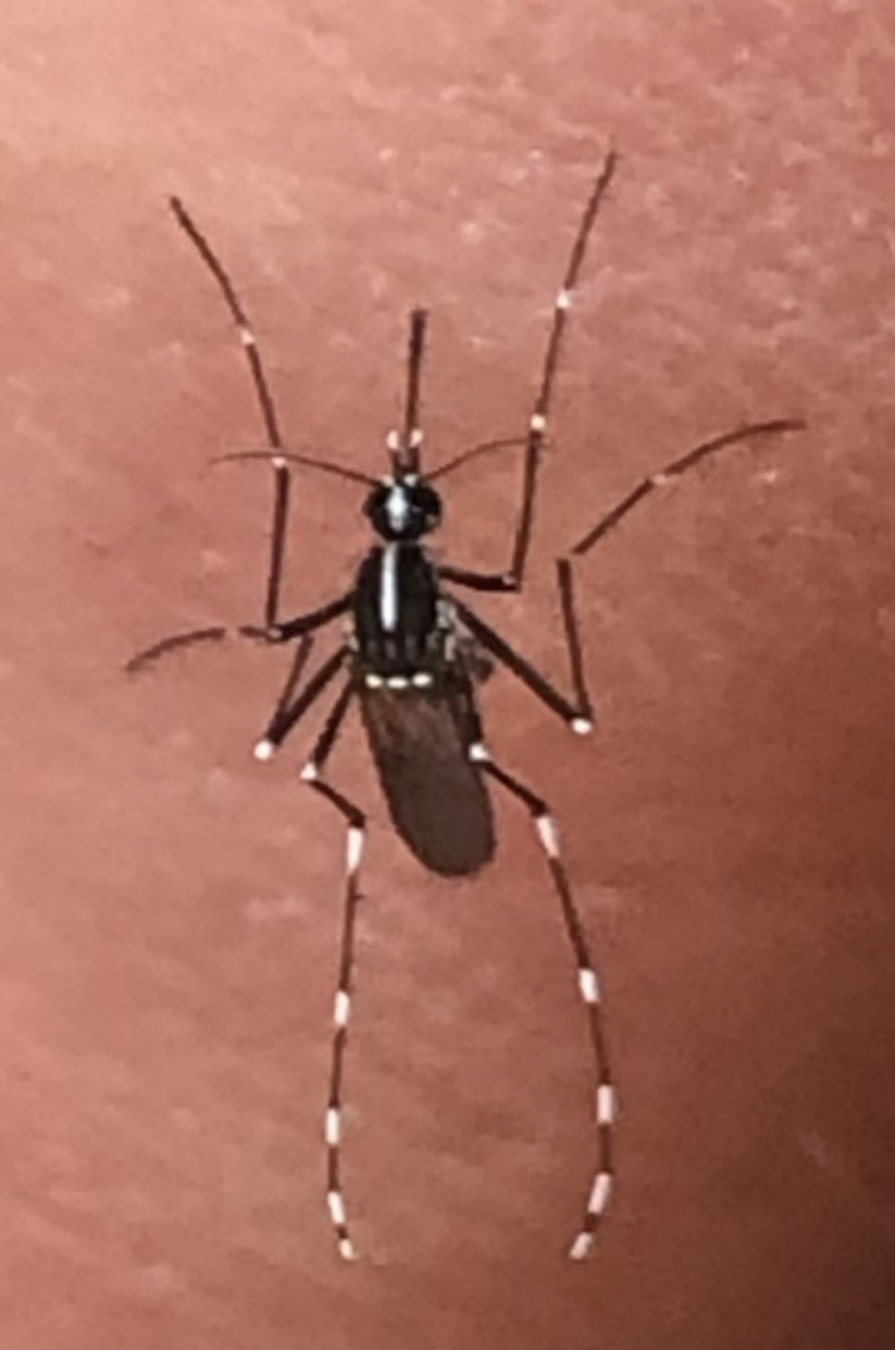
Adult *Ae. albopictus* specimen collected and photographed by citizen scientists in the allotment garden in Liebenau, Graz, Austria (location A)

Citizen scientists collected 14 adult mosquitoes at the allotment garden, which were identified as *Ae. albopictus*. Haplotype analysis of 7 specimens revealed that all specimens belong to haplotype 3 (HPT3) which had been documented in Vienna in the summer of 2020 (Bakran-Lebl et al. 2021) (Fig. 2).

Here, we report the presence of *Ae. albopictus* in Graz, the second largest city of Austria. Initially, a citizen of Graz informed experts of possible tiger mosquitoes in an allotment garden. Subsequently, citizen scientists caught adult mosquitoes and operated ovitraps to further investigate the mosquito situation in the city. This highlights the importance and advantage of citizen science projects concerning mosquito detection and monitoring, as has also been demonstrated during other studies (Bakran-Lebl et al. 2021; Juznic-Zonta et al. 2022).

In the allotment garden (location A), *Ae. albopictus* eggs were found repeatedly throughout the study period. In contrast, at location B (AGES Institute of Medical Microbiology and Hygiene Graz), eggs of *Ae. albopictus* were only detected once in mid-June. Therefore, monitoring efforts need to be continued to confirm the presence and possible establishment of the tiger mosquito at this site.

The presence of eggs and adult specimens in the current study period suggests that the tiger mosquito is already breeding in the allotment garden in Liebenau. However, whether stable populations of tiger mosquitoes can survive in Graz still needs to be investigated. With over 25 allotment garden complexes, Graz supplies *Aedes* mosquitoes with the perfect environment for reproduction, as small artificial water bodies, such as rain barrels, can frequently be found there (Becker et al. 2017; Heimgärtner-Steiermark 2022). Additionally, urban heat islands and the mild climate in the city might also favour the survival of these mosquitoes., Establishment of *Ae. albopictus* in Graz is therefore considered to be highly likely. Furthermore, haplotype analysis revealed that all adult mosquitoes belonged to the same haplotype (HPT3). Although the sample size is too small to make a firm conclusion, the data indicates a single introduction in the allotment garden. If the same haplotype can be found in the following years, this might point to an overwintering population. Several *Ae. albopictus* of haplotype 3 have also been reported in Vienna, the capital city and largest city of Austria, in 2020 (Bakran-Lebl et al. 2021) and in Tyrol (Fuehrer et al. 2020).

*Aedes albopictus* is able to transmit various pathogens, most importantly dengue, Zika and chikungunya viruses (Gloria-Soria et al. 2020; Paixao et al. 2018). The establishment of this species in Austria increases the risk of autochthonous infections and subsequent outbreaks of those pathogens. In Graz, one of the early signs of the presence of alien *Aedes* species were the allotment garden residents’ complaints concerning mosquitoes. This again shows that *Ae. albopictus* (and other alien *Aedes* species) are not only important with respect to their potential to transmit pathogens, but also affect the quality of life of the population, as they are generally considered a daytime nuisance biter (Ravasi et al. 2021; Unlu et al. 2021).

Monitoring efforts in Graz need to continue in order to determine whether stable populations of *Ae. albopictus* are forming or have already been formed. Moreover, it is vital for public health and the quality of life of the inhabitants to raise awareness of the threat that potentially invasive mosquitoes pose, and to establish measures to limit their spread, not only in Graz, but in all of Austria.

## Data Availability

The datasets generated during and/or analysed during the current study are available from the corresponding author upon reasonable request.
